# Effectiveness of medical treatment for Cushing’s syndrome: a systematic review and meta-analysis

**DOI:** 10.1007/s11102-018-0897-z

**Published:** 2018-05-31

**Authors:** Leonie H. A. Broersen, Meghna Jha, Nienke R. Biermasz, Alberto M. Pereira, Olaf M. Dekkers

**Affiliations:** 1 Department of Medicine, Division of Endocrinology, Leiden University Medical Centre, Albinusdreef 2, 2333 ZA Leiden, The Netherlands; 2 Center for Endocrine Tumors Leiden (CETL), Leiden University Medical Center, Albinusdreef 2, 2333 ZA Leiden, The Netherlands; 3 Department of Endocrinology, Diabetes and Nutrition, Charité Universitätsmedizin Berlin, Chariteplatz 1, 10117 Berlin, Germany; 4 Department of Clinical Epidemiology, Leiden University Medical Center, Albinusdreef 2, 2333 ZA Leiden, The Netherlands

**Keywords:** Cushing’s syndrome, Cushing’s disease, Medical treatment, Effectiveness, Side effects

## Abstract

**Purpose:**

To systematically review the effectiveness of medical treatment for Cushing’s syndrome in clinical practice, regarding cortisol secretion, clinical symptom improvement, and quality of life. To assess the occurrence of side effects of these medical therapies.

**Methods:**

Eight electronic databases were searched in March 2017 to identify potentially relevant articles. Randomized controlled trials and cohort studies assessing the effectiveness of medical treatment in patients with Cushing’s syndrome, were eligible. Pooled proportions were reported including 95% confidence intervals.

**Results:**

We included 35 articles with in total 1520 patients in this meta-analysis. Most included patients had Cushing’s disease. Pooled reported percentage of patients with normalization of cortisol ranged from 35.7% for cabergoline to 81.8% for mitotane in Cushing’s disease. Patients using medication monotherapy showed a lower percentage of cortisol normalization compared to use of multiple medical agents (49.4 vs. 65.7%); this was even higher for patients with concurrent or previous radiotherapy (83.6%). Mild side effects were reported in 39.9%, and severe side effects were seen in 15.2% of patients after medical treatment. No meta-analyses were performed for clinical symptom improvement or quality of life due to lack of sufficient data.

**Conclusions:**

This meta-analysis shows that medication induces cortisol normalization effectively in a large percentage of patients. Medical treatment for Cushing’s disease patients is thus a reasonable option in case of a contraindication for surgery, a recurrence, or in patients choosing not to have surgery. When experiencing side effects or no treatment effect, an alternate medical therapy or combination therapy can be considered.

**Electronic supplementary material:**

The online version of this article (10.1007/s11102-018-0897-z) contains supplementary material, which is available to authorized users.

## Introduction

Cushing’s syndrome due to endogenous glucocorticoid excess is either adrenocorticotropic hormone (ACTH)-dependent or ACTH-independent, both with a variety of underlying causes [1]. Cushing’s disease results from an ACTH-secreting pituitary adenoma and has a reported incidence of approximately 1.2–2.4 patients per million each year [2]. Ectopic Cushing’s syndrome is a rare condition resulting from a non-pituitary ACTH-producing source. ACTH-independent Cushing’s syndrome is caused by a cortisol-producing adrenal adenoma or carcinoma [1]. Excess of glucocorticoids alters body composition and metabolic profile, inducing fat maldistribution, muscle wasting, insulin resistance, dyslipidemia, hypercoagulability, and increasing the risk of osteoporosis, hypertension, and neuropsychiatric disorders [3, 4].

Transsphenoidal pituitary adenomectomy is a well-established and effective treatment for Cushing’s disease [5]. Cushing’s syndrome is generally approached by removing the ACTH-producing tumor in ectopic Cushing’s syndrome and by adrenalectomy in ACTH-independent Cushing’s syndrome [6]. However, there is increasing experience with first line medical treatment, both for patients with contraindications for surgery and for patients with recurrent disease [7]. Furthermore, drugs can be used to control cortisol secretion preoperatively and to bridge the time period until control of hypercortisolism is achieved by radiotherapy [7]. Drugs used in medical practice vary per country and underlying cause of Cushing’s syndrome and include ketoconazole, metyrapone, mitotane, cabergoline, pasireotide, and mifepristone [7, 8]. A recent review described the percentage of patients achieving cortisol normalization after monotherapy with the steroidogenesis inhibitors ketoconazole and metyrapone [9]. However, until now no systematic review and meta-analysis has been performed to summarize the effectiveness of all medical agents used in clinical practice (ketoconazole, metyrapone, mitotane, cabergoline, pasireotide, and mifepristone).

### Study aims

The primary aim of the present systematic review and meta-analysis was to evaluate the effectiveness of medical treatment for Cushing’s syndrome in clinical practice. Effectiveness of medical treatment was evaluated regarding cortisol secretion, clinical symptom improvement, and quality of life. The secondary study aim was to compare these medical therapies according to occurrence of side effects.

## Methods

### Eligibility criteria

Randomized controlled trials and cohort studies assessing the effectiveness of FDA/EMA approved medical treatment for treatment of Cushing’s syndrome, either de novo or with persistent or recurrent disease, were eligible for inclusion, as well as cabergoline, which has been used for Cushing’s syndrome in multiple investigator initiated clinical trials. Medical agents considered were ketoconazole, metyrapone, mitotane, cabergoline, pasireotide, and mifepristone. Articles were excluded if reporting broad inclusion categories without separating the subgroup of Cushing’s patients, or if the study included children (age < 18 years) only. For eligibility, at least ten patients had to be included per treatment group to minimize risk of selection bias. For multiple articles describing (partially) overlapping populations, the article with the largest study population was included in the analysis. Articles irretrievable online were requested by contacting the authors. Only articles in the English language were considered.

### Search strategy

To identify potentially relevant articles, PubMed, Embase, Web of Science, COCHRANE Library, CENTRAL, Emcare, LWW and ScienceDirect were systematically searched in March 2017 in cooperation with a specialized librarian (see Online Resource 1 for the complete search strategy). The search was repeated in PubMed in May 2017. Furthermore, references of included articles were searched to increase the number of potentially eligible articles.

### Data extraction

All identified articles were imported in EndNote 8 (Thomson Reuters, Philadelphia, PA, USA). Studies were screened by title and abstract and two independent reviewers reviewed potentially relevant articles in detail. The Meta-analysis Of Observational Studies in Epidemiology (MOOSE) guidelines were used for reporting [10].

From included articles we extracted the following data: number and type of Cushing’s syndrome patients included, type of medical agent used, treatment dose, treatment duration, duration of follow-up, number of patients pre-treated with medication before surgery, number of patients with normalization of cortisol, clinical improvement, well-being, quality of life, and side effects. Where available, separate outcomes were extracted for patients with primary treatment (before any other treatment for Cushing’s syndrome) and patients with secondary treatment (after recurrence or failure of surgery and/or radiotherapy). For clinical improvement, all reported symptoms as well as general statements (e.g. “Clinical signs regressed in full responders”, without specifying which clinical signs) were extracted. However, only hypertension and diabetes mellitus were considered for analysis, because these symptoms were expected to be reported homogeneously by multiple articles. For quality of life, all general and Cushing’s disease specific questionnaires were considered. All reported side effects were extracted.

### Risk of bias assessment

We used a component approach to assess risk of bias for all included studies. The following components were included, which could potentially bias a reported association between medical treatment for Cushing’s syndrome and outcome:

1. Inclusion of patients (consecutive inclusion from all patients eligible or a random sample is considered low risk of bias)
2. Loss to follow-up (< 5% is considered low risk of bias)
3. Criteria for diagnosis of Cushing’s syndrome adequately reported (see below)
4. Outcome measurement for cortisol normalization: urinary free cortisol, midnight salivary cortisol or a low dose dexamethasone test is considered low risk of bias
5. Reporting of outcome definition (see below)
6. Description of protocol for laboratory measurements (see below)
7. Description of dose and duration of intervention (see below)

As criteria for diagnosis of Cushing’s syndrome vary widely over time and even by study center, and per underlying etiology, adequately reporting the criteria used for diagnosis is considered a low risk of bias. Reporting of outcome definition is considered adequate if the article at least mentioned which outcome was studied, which test was used to determine the outcome, and if applicable, which cortisol level had to be measured. Description of protocol for laboratory measurements is considered adequate if the assay used for measuring cortisol is reported, or the assay for the main outcome if this was not cortisol. Description of dose and duration of intervention is considered adequate if dose (per day or per week) and duration of medical treatment are reported (average and range, or exact dose and duration if this is equal for all patients). Also considered adequate is reporting of exact treatment protocol for trials with dose increase based on cortisol levels. Referring to another published article in which the information is reported is also considered adequate.

Risk of bias assessment was conducted to explore potential heterogeneity. As there were no studies that compared two different medical agents directly, confounding was not judged at the study level, but was assessed by comparing baseline characteristics for all included studies.

### Study endpoints

Primary outcome of this study was the effectiveness of medical treatment for Cushing’s syndrome, represented by the pooled percentage of patients reaching normalization of cortisol secretion (definition according to the authors) after medical treatment, patients showing symptom improvement, improved well-being and improved quality of life. Main analyses were performed in studies reporting on pituitary Cushing patients. Separate analyses were performed for publications reporting on (1) mixed etiologies other than adrenocortical carcinomas, (2) mixed etiologies including adrenocortical carcinomas, and (3) ectopic Cushing only, if sufficient data were available. Studies were categorized according to type of medical agent (ketoconazole, metyrapone, mitotane, cabergoline, pasireotide, and mifepristone). Studies using more than one of the above mentioned medical agents at the same time or consecutively were assessed separately.

For normalization of cortisol, all measurements of cortisol were considered. However, measurement of urinary free cortisol, midnight salivary cortisol or morning cortisol after a low dose dexamethasone test was considered low risk of bias (see above). Data on reduction of cortisol as a percentage of baseline were not considered for analysis.

Secondary outcomes were the pooled percentages of patients with mild or severe side effects stratified by medical agent. Severe side effects were considered those that required therapy adjustment or withdrawal, as well as all side effects categorized as severe by the authors. Mild side effects were all not categorized as severe. For articles reporting only specific side effects, the side effect that affected the most patients was included in the analysis.

Subgroup analyses were performed according to indication (primary therapy, including pre-treatment before surgery, and therapy for recurrence) if described separately. As few studies provided separate data for primary/secondary analysis, a separate subgroup analysis was performed, in which studies were categorized as low (≤ 20%) or high (≥ 80%) percentage of patients using medical agents as pre-treatment before surgery. A separate analysis was performed for normalization of cortisol according to the presence of multiple medical treatments and concurrent or previous radiotherapy.

### Statistical analysis

A random-effects logistic regression model was used to pool percentages for analyses including ≥ 5 articles, whereas a fixed-effects logistic regression model was used for analyses including < 5 articles. All pooled percentages are accompanied by 95% confidence intervals. The Freeman-Tukey arcsine transformation was used to stabilize variances, in order to prevent exclusion of studies with 0 or 100% as outcome. All analyses were performed using Stata 11.2 (Stata Corp., College Station, TX, USA).

Sensitivity analyses were performed for normalization of cortisol for low risk of bias studies, and for the combination of low and intermediate risk of bias studies. Articles were considered low risk of bias if they adhered to at least six (out of seven) of the above mentioned criteria for risk of bias. Only one article adhered to all seven criteria [11]. Articles were considered intermediate risk of bias if they adhered to five of the above mentioned criteria for risk of bias.

## Results

### Study selection

**Fig. 1:**
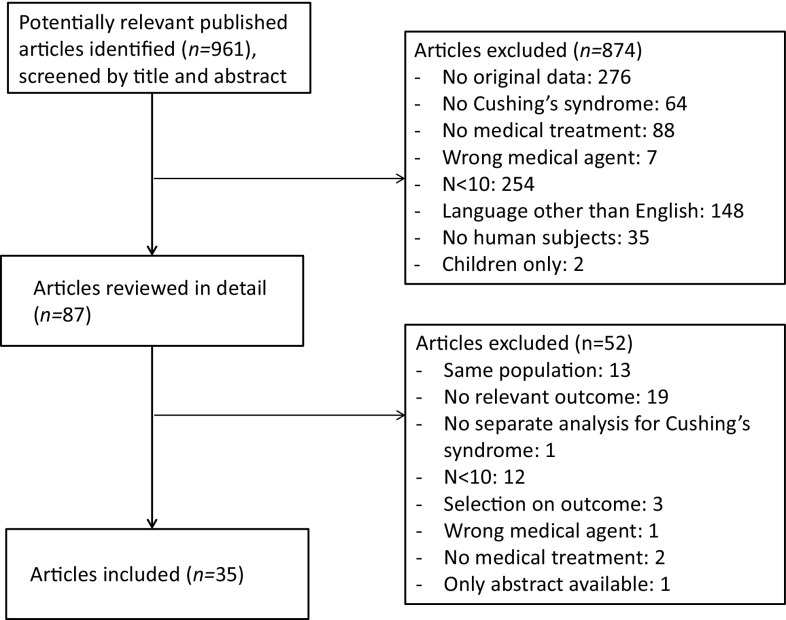
Flow-chart of inclusion of articles in this systematic review

The initial search identified 960 potentially relevant articles. Searching through references of included articles identified one additional article, thereby yielding a total of 961 articles. By screening these articles by title and abstract, 874 articles were excluded. The remaining 87 articles were reviewed in detail. Reasons for exclusion are summarized in Fig. 1. In total, 35 articles were included, reporting on six different drugs.

### Study characteristics (Online Resource 2)

We included five studies on mitotane [12–16], two on pasireotide [17, 18], three on cabergoline [19–21], eight on ketoconazole [22–29], five on metyrapone,[30–34] two on mifepristone [35, 36], and ten on multiple medical agents [11, 37–45]. Studies were published between 1971 and 2017. There were eleven single-arm trials, two randomized trials with two treatment arms (pasireotide 600 µg vs. pasireotide 900 µg; first cabergoline, then add ketoconazole vs. first ketoconazole, then add cabergoline), and 22 cohort studies. We included eighteen studies on pituitary Cushing’s disease only, two on ectopic Cushing’s syndrome only, and fifteen on Cushing’s syndrome patients due to various underlying etiologies. In total, 1520 patients were included. There were 28 articles measuring normalization of cortisol by at least urinary free cortisol, midnight salivary cortisol or a low dose dexamethasone test (88% of 32 articles reporting normalization of cortisol as an outcome). There were 25 studies reporting on clinical improvement, and three studies reporting on quality of life.

Baseline characteristics of included studies show clear differences between studies. Reported average age varied between 32.2 and 60.0 years. Percentage female varied between 21.7 and 95.0%. Average duration of follow-up was 2 weeks to 11.5 years. Seven articles reported that at least part of their study population received radiotherapy in addition to medical treatment (two with mitotane, one with ketoconazole, and four with metyrapone). Pasireotide and cabergoline were used only for pituitary Cushing’s disease, whereas all other medical agents were used for various etiologies of Cushing’s syndrome.

### Risk of bias assessment (Online Resource 3)

Loss to follow-up [reported in twelve studies (34%)] ranged from 0 to 60%. Inclusion of consecutive patients or a random sample was explicitly stated in seventeen articles (49%). Criteria for diagnosis of Cushing’s syndrome were adequately reported in 31 articles (89%). Reporting of outcome definition was adequate in 25 articles (71%). Description of protocol for laboratory measurements was adequate in 23 articles (66%). Description of dose and duration of intervention was adequate in 23 articles (66%). A total of nine articles were with low risk of bias (adherent to at least six out of seven criteria) and another nine articles with intermediate risk of bias (adherent to five out of seven criteria).

### Study outcomes

For 26 articles (1000 patients) normalization of cortisol was reported as outcome measure. There were 25 articles reporting on clinical improvement. One article reported no improvement in any of the measured clinical symptoms (weight, blood pressure, glucose and HbA1c) [19]. All other articles reported improvement in one or more clinical symptoms. Well-being was not reported by any of the included articles. Three articles (228 patients) reported on quality of life. These reported an improvement in CushingQoL score, SF-36 score, and emotional reaction on the Nottingham Health Profile (NHP), and on the other hand more pain measured by the RAND-36 [18, 36, 44]. There were 30 articles (86%) reporting at least one side effect. Two of these articles, using mifepristone, described an increase in cortisol levels during the study period in a total of 47 out of 70 included patients (67%) [35, 36]. No meta-analysis was performed for clinical improvement, as results were considered too heterogeneous. Hypertension and diabetes mellitus were described by heterogeneous articles (type of medical agent, etiology of Cushing’s syndrome) and the type of outcome was heterogeneous (difference in blood pressure and glucose vs. number of patients with improved values, only patients with disturbed values at start of the study vs. all patients analysed). Detailed study outcomes at the individual study level are reported in Online Resource 4.

### Meta-analyses of normalization of cortisol (Table 1; Fig. 2: normalization of cortisol per medical agent in pituitary Cushing)

**Table 1:** Results of meta-analyses according to etiology of Cushing’s syndrome

	Pituitary Cushing’s disease		All etiologies (pituitary, adrenal, ectopic) other than adrenal carcinoma		All etiologies (pituitary, adrenal, ectopic) including adrenal carcinoma		Ectopic Cushing’s syndrome	
	Estimated percentage	95% confidence interval (N)	Estimated percentage	95% confidence interval (N)	Estimated percentage	95% confidence interval (N)	Estimated percentage	95% confidence interval (N)
Normalization of cortisol	59.5	48.4–70.1 (18)	61.6	51.8–71.0 (22)	64.7	55.6–73.3 (26)	83.3	64.9–96.8 (3)
Per medical agent								
Mitotane	81.8	75.4–87.6 (4)	81.8	75.4–87.6 (4)	79.8	73.3–85.7 (4)	–	–
Pasireotide	41.1	32.7–49.8 (2)	41.1	32.7–49.8 (2)	41.1	32.7–49.8 (2)	–	–
Cabergoline	35.7	24.6–47.6 (3)	35.7	24.6–47.6 (3)	35.7	24.6–47.6 (3)	–	–
Ketoconazole	49.0	42.0–56.0 (3)	49.3	42.6–56.0 (4)	71.1	51.6–87.5 (7)	–	–
Metyrapone	60.0^a^	31.3–83.2^a^	75.9	57.5–90.9 (2)	75.9	57.5–90.9 (2)	–	–
Mifepristone	–	–	–	–	–	–	–	–
Multiple medical agents	65.7	46.9–82.4 (5)	67.8	51.9–81.9 (7)	67.6	53.6–80.3 (8)	–	–
Primary treatment	58.1	49.7–66.2 (4)	49.4	41.3–57.5 (4)	49.4	41.3–57.5 (4)	–	–
Secondary treatment	57.8	41.3–73.6 (5)	48.6	41.2–56.1 (4)	48.6	41.2–56.1 (4)	–	–
Per percentage pretreatment								
≤20%	59.7	49.4–69.6 (8)	59.7	49.4–69.6 (8)	59.7	49.4–69.6 (8)	–	–
≥80%	32.3	20.0–45.8 (2)	42.6	33.5–51.9 (3)	53.6	45.0–62.0 (4)	–	–
Adjuvant treatment								
No other treatment	49.4	36.0–62.9 (10)	52.7	40.1–65.1 (12)	57.2	44.4–69.6 (14)	–	–
Multiple medical agents	65.7	46.9–82.4 (5)	67.8	51.9–81.9 (7)	67.6	53.6–80.3 (8)	–	–
Radiotherapy	83.6	75.5–90.4 (3)	83.6	75.5–90.4 (3)	84.8	78.0–90.6 (4)	–	–
Sensitivity analysis (low risk of bias)								
Mitotane	71.6^a^	59.9–81.0^a^	71.6^a^	59.9–81.0^a^	71.6^a^	59.9–81.0^a^		–
Pasireotide	17.2^a^	7.6–34.5^a^	17.2^a^	7.6–34.5^a^	17.2^a^	7.6–34.5^a^	–	–
Cabergoline	35.0	20.6–50.9 (2)	35.0	20.6–50.9 (2)	35.0	20.6–50.9 (2)	–	–
Ketoconazole	–	–	–	–	–	–	–	–
Metyrapone	–	–	–	–	–	–	–	–
Mifepristone	–	–	–	–	–	–	–	–
Multiple medical agents	50.6	40.9–60.2 (3)	50.6	40.9–60.2 (3)	50.6	40.9–60.2 (3)	–	–
Sensitivity analysis (low and intermediate risk of bias)								
Mitotane								
Pasireotide	71.6^a^	59.9–81.0^a^	71.6^a^	59.9–81.0^a^	71.6^a^	59.9–81.0^a^	–	–
Cabergoline	17.2^a^	7.6–34.5^a^	17.2^a^	7.6–34.5^a^	17.2^a^	7.6–34.5^a^	–	–
Ketoconazole	35.7	24.6–47.6 (3)	35.7	24.6–47.6 (3)	35.7	24.6–47.6 (3)	–	
Metyrapone	48.5^a^	41.7–55.4^a^	48.3	41.5–55.2 (2)	59.8	54.1–65.4 (4)		
Mifepristone	–	–	83.3^a^	60.8–94.2^a^	83.3^a^	60.8–94.2^a^	–	
Multiple medical agents	53.1	43.8–62.3 (4)	67.5	46.5–85.7 (5)	67.4	50.0–82.8 (6)		
Mild side effects	39.9	25.0–55.8 (13)	40.2	27.4–53.8 (15)	35.3	25.6–45.7 (23)	–	–
Per medical agent								
Mitotane	68.5	59.1–77.2 (3)	68.5	59.1–77.2 (3)	69.1	60.0–77.6 (3)	–	–
Pasireotide	58.3	51.4–65.1 (2)	58.3	51.4–65.1 (2)	58.3	51.4–65.1 (2)	–	–
Cabergoline	24.0	14.4–35.1 (3)	24.0	14.4–35.1 (3)	24.0	14.4–35.1 (3)	–	–
Ketoconazole	–	–	–	–	22.6	15.1–31.0 (3)	–	–
Metyrapone	30.8^a^	12.7–57.6^a^	32.2	16.3–50.3 (2)	19.7	12.2–28.2 (3)	–	–
Mifepristone	–	–	–	–	35.6	24.5–47.4 (2)	–	–
Multiple medical agents	18.0	10.6–26.7 (4)	25.5	7.6–48.5 (5)	26.7	12.8–43.2 (7)	–	–
Severe side effects	15.2	9.1–22.4 (12)	16.2	10.1–23.3 (14)	15.3	10.1–21.3 (21)	–	–
Per medical agent								
Mitotane	28.4^a^	19.0–40.1^a^	28.4^a^	19.0–40.1^a^	28.4^a^	19.0–40.1^a^	–	–
Pasireotide	15.7	10.9–21.2 (2)	15.7	10.9–21.2 (2)	15.7	10.9–21.2 (2)	–	–
Cabergoline	4.8	0.5–11.9 (3)	4.8	0.5–11.9 (3)	4.8	0.5–11.9 (3)	–	–
Ketoconazole	20.5^a^	15.5–26.6^a^	18.8	13.5–24.6 (2)	14.2	10.3–18.7 (4)	–	–
Metyrapone	7.7^a^	1.4–33.3^a^	27.1	12.2–44.7 (2)	16.2	9.4–24.3 (3)	–	–
Mifepristone	–	–	–	–	42.0	30.4–54.0 (2)	–	–
Multiple medical agents	20.9	13.6–29.2 (4)	20.9	13.6–29.2 (4)	14.6	4.2–28.8 (6)	–	–
Result of severe side effect								
Adjust therapy	23.9	15.9–32.8 (4)	23.6	10.0–40.4 (6)	20.4	8.6–35.2 (9)	–	–
Stop therapy	8.5	2.8–16.3 (9)	8.5	2.8–16.3 (9)	8.5	4.6–13.4 (15)	–	–

*N* number of articles included in meta-analysis

^a^No meta-analysis was performed, as the total number of studies equaled 1 (results only shown for analyses per medical agent if other medical agents were reported in multiple articles)

**Fig. 2:**
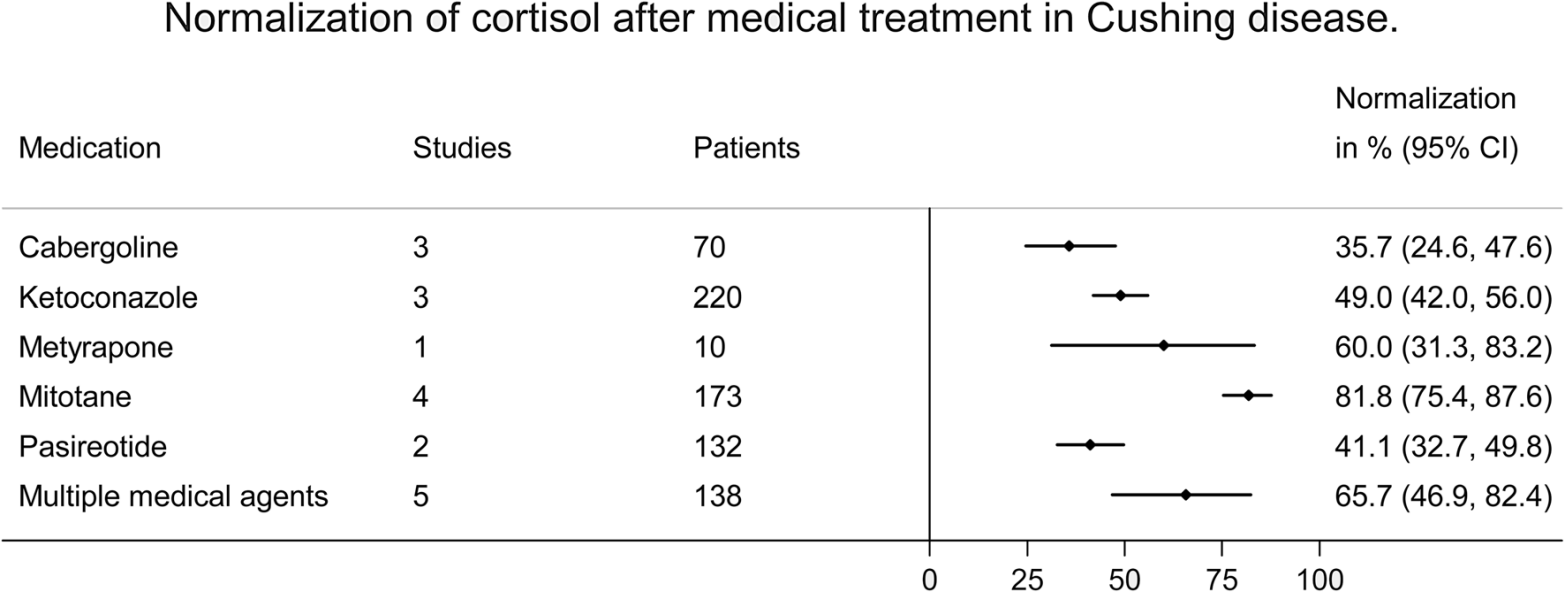
Meta-analysis of normalization of cortisol after medical treatment in Cushing disease

For Cushing’s disease, pooled reported treatment effect ranged from 32.3% if a large percentage of patients used medication as pre-treatment before surgery, to 83.6% if medication was combined with radiotherapy. When comparing medical agents, a relatively high percentage of patients using mitotane showed normalization of cortisol (81.8%), whereas treatment with cabergoline and pasireotide less often normalized cortisol secretion (35.7 and 41.1%). For detailed results, including data on different etiologies (mixed etiologies other than adrenocortical carcinomas, mixed etiologies including adrenocortical carcinomas, and ectopic Cushing), see Table 1.

Seven studies reported data separately for medication as primary (n = 4) and/or secondary therapy (n = 5). For patients with pituitary Cushing’s disease, medication as primary therapy normalized cortisol in 58.1% (95% CI 49.7–66.2%), similar to the effect of medication as secondary therapy, 57.8% (95% CI 41.3–73.6%). Articles in which ≤ 20% of patients were medically pre-treated before surgery showed normalization of cortisol in 59.7% of patients (95% CI 49.4–69.6%). Articles in which ≥ 80% of patients were pre-treated with medication before surgery showed a preoperative normalization of cortisol in 32.3% (95% CI 20.0–45.8%) for patients with pituitary Cushing’s disease. Patients with medical monotherapy showed a relatively low percentage of cortisol normalization. This percentage was higher for patients using multiple agents, and highest for patients with concurrent or previous radiotherapy.

The sensitivity analyses, both excluding articles with high risk of bias (n = 18 included), as well as excluding articles with high and intermediate risk of bias (n = 9 included), showed similar results as the main analysis. The most remarkable difference is that lower percentages of patients with normalization of cortisol were seen for multiple medical agents in both sensitivity analyses than for multiple medical agents in the main analysis.

### Meta-analyses of side effects (Table 1; Fig. 3: side effects per medical agent in pituitary Cushing)

**Fig. 3:**
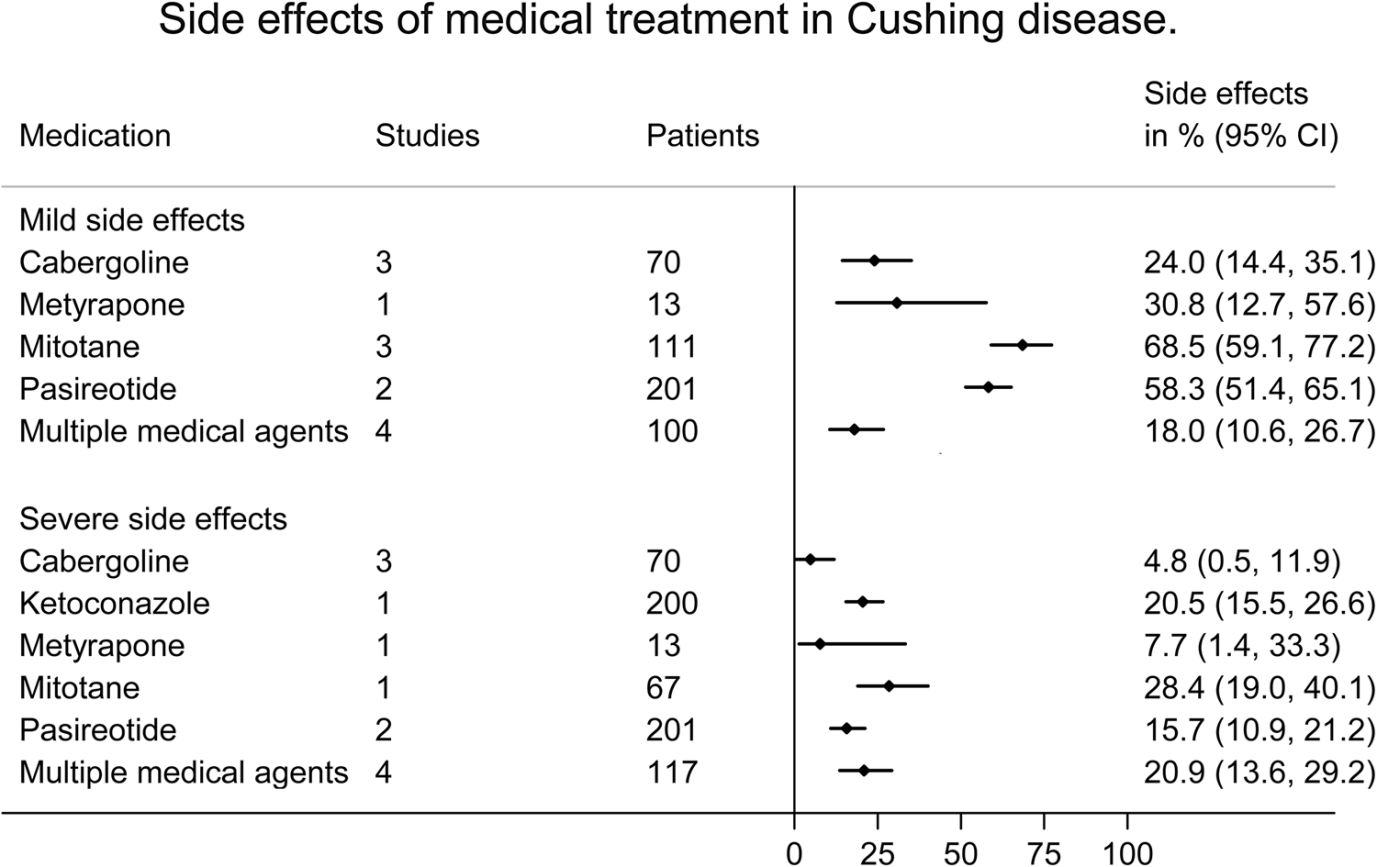
Meta-analysis of side effects after medical treatment in Cushing disease

For pituitary Cushing’s disease, mild side effects were reported in 39.3% (95% CI 25.0–55.8%) of patients after medical treatment. Patients using mitotane and pasireotide more often showed mild side effects compared to the total population (68.5 and 58.3%).

For pituitary Cushing’s disease, severe side effects were seen in 15.2% (95% CI 9.1–22.4%). In the group of mixed etiologies including adrenocortical carcinoma, patients using mifepristone showed severe side effects relatively often (42.0%; 95% CI 30.4–54.0%). Due to the severe side effects, 23.9% (95% CI 15.9–32.8%) of patients adjusted their medical therapy, and 8.5% (95% CI 2.8–16.3%) of patients stopped their medical therapy.

## Discussion

We performed a systematic review and meta-analysis to evaluate effectiveness of medical treatment in routine clinical practice in Cushing’s syndrome. Medical treatment was effective in normalizing cortisol levels in Cushing’s syndrome in 35.7% (cabergoline) to 81.8% (mitotane) of patients. Furthermore, the combined use of medical agents at the same time or consecutively increased the percentage of patients with normalized cortisol secretion (65.7%). Importantly, medical agents for hypercortisolism can cause severe side effects, leading to therapy adjustment or withdrawal in 4.8% (cabergoline) to 28.4% (mitotane) of patients. These results suggest that medical therapy can be considered a reasonable treatment alternative to the first choice surgical treatment when regarding treatment effectiveness and side effects.

This study is the first systematic review and meta-analysis of all medical agents currently used in clinical practice for Cushing’s syndrome. Only one previous study performed a systematic review and meta-analysis of two medical agents in Cushing’s syndrome. Daniel et al. studied normalization of cortisol after monotherapy with ketoconazole or metyrapone, and found that urinary free cortisol normalized in 60% (ketoconazole) and that normalization of hypercortisolism as defined by the authors occurred in 75% of patients using metyrapone [9]. This is in line with the results obtained in the current study (49.0–71.1% for ketoconazole and 60.0–75.9% for metyrapone depending on patient categories). Since our last search in May 2017, one more research article on medical treatment in Cushing’s syndrome was published. Lacroix et al. described a 12 month clinical trial using pasireotide in various dosages in 150 patients. Urinary free cortisol normalized in 41.3% of patients. This is in line with our own results for cortisol excretion normalization for patients using pasireotide (41.1%). Adverse events grade 1–2 were described in up to 48% of patients, and adverse events grade 3–4 in up to 16% of patients. This is in line with our own results for mild side effects (58.3%) and severe side effects (15.7%) for patients using pasireotide [46].

In interpreting the results, the following study limitations need to be taken into account. There was a large amount of heterogeneity in included studies, regarding medical agent, etiology of Cushing’s syndrome, indication for use of medical therapy for Cushing (presurgical cortisol control, contra-indication for surgery, post-surgical therapy failure or recurrence), outcome measurement (definition of cortisol normalization), and concurrent or previous use of radiotherapy. Heterogeneity concerning medical agent and etiology was handled by performing separate analyses per medical agent and per etiology, although this substantially reduced the number of articles in some categories. Due to heterogeneity regarding outcome measurement for clinical symptoms (difference in blood pressure and glucose vs. number of patients with improved values, only patients with disturbed values at start of the study vs. all patients analysed), we were unable to perform a quantified analysis of clinical improvement after medical therapy. Included articles showed various levels of risk of bias. However, sensitivity analyses excluding articles with high risk of bias or with high and intermediate risk of bias showed similar results to the main analysis.

For patients with pituitary Cushing’s disease, first line transsphenoidal surgery yields better results than medical therapy (80% remission). However, remission after a repeat surgery was shown to be only 42.6–55.7% in a recent meta-analysis. Various complications occurred in up to 18.5% of patients [47]. This underlines that medical treatment is a reasonable alternative to repeat surgical procedure.

Differences in effectiveness and side effects between the various etiological groups were small. In ectopic Cushing’s syndrome only, a higher percentage of patients reached normalization of cortisol levels than in the other etiological categories. As there were only three articles with separate data on patients with ectopic Cushing’s syndrome (of which one article only reported on one patient with ectopic Cushing’s syndrome, besides reporting on Cushing patients with other etiologies), no further subanalyses were possible, and reliability of this result is uncertain. However, we found no explanation for this high percentage of cortisol normalization in ectopic Cushing’s syndrome when considering risk of bias, type of medical agent, or additional treatment.

When comparing different medical agents, it seems that a high percentage of patients with cortisol normalization corresponds to a high percentage of patients with side effects and vice versa. However, all dosages used and studied were within the boundaries advised by the European Medicines Agency (EMA) and the food and drug administration (FDA). It might mean that advised and commonly used dosages are in fact not the optimal dosages for treatment of Cushing’s syndrome considering the balance between treatment effect and side effects. Furthermore, medical agents with a high percentage of patients with normalized cortisol secretion (mitotane and metyrapone) are relatively often combined with radiotherapy, which may lead to overestimating the effect and side effects of the drug per se. The use of combined medical therapy, in combination or consecutively, increases the likelihood of successful treatment, i.e. in normalizing cortisol levels. This suggests that sensitivity for different medical agents may vary per patient.

No difference was shown in normalization of cortisol between patients using medical agents as primary treatment vs. secondary treatment, suggesting that effectiveness of medical agents is independent of other treatment modalities. A higher percentage of patients reaches normalization of cortisol in studies where a small part of included patients received medication preoperatively (including patients with contraindications to surgery and patients after previous surgery) than in studies where a large part of included patients received medication before elective surgery. The most likely explanation is that in patients with planned surgery, medication is given to control cortisol excess, and surgery is performed before complete normalization of cortisol occurs. Unfortunately, we don’t know the average time until cortisol normalization, as this was not reported by most articles. Total follow-up time was not different for studies with a high percentage of medical pre-treatment before surgery (average 0.1–11.5 years) than for all included studies. It would be interesting to know if the effectiveness of surgery is dependent upon the normalization of cortisol with medical treatment before surgery compared to presurgical medical treatment without cortisol normalization and compared to no presurgical medical treatment at all. Especially in pituitary Cushing’s disease, the percentage of patients with preoperative medical treatment that reaches cortisol normalization is low. This might be due to the higher expectations of surgery in pituitary Cushing’s disease compared to other etiologies, which may be why surgery is performed before patients reach normalization of cortisol levels. However, these results should be interpreted with caution, as a small number of articles were included in these analyses, and in the articles with a mixed etiologies population, a large proportion of included patients had pituitary Cushing’s disease [23, 43].

Based on the current study, medication can be regarded a valuable alternative to pituitary surgery for Cushing’s disease patients with contraindications to surgery, patients with a recurrence considering repeat surgery, and patients that choose not to undergo surgery. For all other Cushing’s disease patients, pituitary surgery remains the first-choice treatment. For other etiologies of Cushing’s syndrome, at present, there is insufficient evidence to recommend when to use medical treatment to lower cortisol levels. However, from the total group of patients, it suggests that medical agents have similar effectiveness in normalizing cortisol levels for all etiologies of Cushing’s syndrome. For a higher chance of treatment success, a different medical agent could be tried if there is no treatment effect or if the patient experiences side effects. There is no evidence for which drug should be used first. Based on the current study, mitotane or metyrapone seem to be most effective in normalizing cortisol levels, but also cause the highest percentage of patients with side effects.

In conclusion, we consider medical treatment for Cushing’s disease a reasonable option in patients with contraindication to surgery, with a recurrence, or that choose not to have surgery. Patients that experience side effects or no treatment effect should be advised to start treatment with a different medical agent to increase the chance of treatment success.

## Electronic supplementary material

Below is the link to the electronic supplementary material.

**Online Resource 1** Search strategy (DOCX 22 KB)

**Online Resource 2** Study characteristics (DOCX 33 KB)

**Online Resource 3** Risk of bias assessment (DOCX 28 KB)

**Online Resource 4** Study outcomes (DOCX 33 KB)
