# Supplementary material for: Effectiveness of medical treatment for Cushing’s syndrome: a systematic review and meta-analysis
Source: Pituitary. 2018 May 31;21(6):631–41. doi: 10.1007/s11102-018-0897-z (PMC6244780; doi:10.1007/s11102-018-0897-z)
Supplement: Supplementary file 1 — Online Resource 1 Search strategy (DOCX 22 KB) [file 11102_2018_897_MOESM1_ESM.docx]

**Pituitary**

**Effectiveness of medical treatment for Cushing’s syndrome – a systematic review and meta-analysis.**

Leonie H.A. Broersen^1,2,3^, Meghna Jha^3^, Nienke R. Biermasz^1,2^, Alberto M. Pereira^1,2^, Olaf M. Dekkers^1,2,4^

^1^Department of Medicine, division of Endocrinology, Leiden University Medical Centre, Albinusdreef 2, 2333 ZA, Leiden, The Netherlands
^2^Center for Endocrine Tumors Leiden (CETL), Leiden University Medical Center, Albinusdreef 2, 2333 ZA, Leiden, The Netherlands  ^3^Department of Endocrinology, Diabetes and Nutrition, Charité Universitätsmedizin Berlin, Chariteplatz 1, 10117, Berlin, Germany

^4^Department of Clinical Epidemiology, Leiden University Medical Center, Albinusdreef 2, 2333 ZA, Leiden, The Netherlands

Corresponding author: L.H.A. Broersen, L.H.A.Broersen@lumc.nl, +31 (0)71-5263082

**Online Resource 1**

1. Search strategy
**PubMed** (<http://www.ncbi.nlm.nih.gov/pubmed?otool=leiden>)
**(("Cushing Syndrome"[Mesh] OR "Cushings Disease"[tw] OR "Cushings Syndrome"[tw] OR "Cushing's Disease"[tw] OR "Cushing's Syndrome"[tw] OR "Cushing Disease"[tw] OR "Cushing Syndrome"[tw] OR Cushing*[tw] OR "Hypercortisolism"[tw] OR Hypercortisol*[tw] OR "Pituitary ACTH Hypersecretion"[Mesh] OR "Pituitary ACTH Hypersecretion"[tw] OR "Inappropriate ACTH Secretion"[tw]) AND ("Ketoconazole"[Mesh] OR "ketoconazole"[tw] OR ketoconazol*[tw] OR "R-41400"[tw] OR "R 41400"[tw] OR "R41,400"[tw] OR "Nizoral"[tw] OR "Metyrapone"[Mesh] OR "Metyrapone"[tw] OR metyrapon*[tw] OR "Methopyrapone"[tw] OR "SU 4885"[tw] OR "Metopirone"[tw] OR "Metopiron"[tw] OR "Métopirone"[tw] OR "Mitotane"[Mesh] OR** **"Mitotane"[tw] OR mitotan*[tw] OR "o,p-DDD"[tw] OR "Chlodithane"[tw] OR "Lysodren"[tw] OR "Chloditan"[tw] OR "Khloditan"[tw] OR "pasireotide"[Supplementary Concept] OR "pasireotide"[tw] OR pasireotid*[tw] OR "SOM-230"[tw] OR "SOM 230"[tw] OR "SOM230"[tw] OR "Mifepristone"[Mesh]** OR **"Mifepristone"[tw] OR mifepriston*[tw] OR "Mifeprex"[tw] OR "Mifegyne"[tw] OR "Mifégyne"[tw] OR "RU-486"[tw] OR "RU 486"[tw] OR "RU486"[tw] OR "R38486"[tw] OR "RU-38486"[tw] OR "RU 38486"[tw] OR "RU38486"[tw] OR "cabergoline"[Supplementary Concept]** OR **"cabergoline"[tw] OR cabergolin*[tw] OR "Galastop"[tw] OR "FCE 21336"[tw] OR "FCE-21336"[tw] OR "Dostinex"[tw]) AND ("cortisol"[tw] OR cortisol*[tw] OR "Hydrocortisone"[Mesh] OR "symptom"[tw] OR "symptoms"[tw] OR symptom*[tw] OR "well being"[tw] OR "wellbeing"[tw] OR "Quality of Life"[Mesh]** OR **"Quality of Life"[tw] OR "QOL"[tw] OR "Hypertension"[mesh] OR hypertens*[tw] OR "Diabetes Mellitus"[mesh] OR diabet*[tw])) NOT ("Animals"[mesh] NOT "Humans"[mesh]) NOT (("case reports"[ptyp] OR "case report"[ti]) NOT ("Clinical Study"[ptyp] OR "series"[ti] OR "review"[ptyp] OR "review"[ti]))**

**Embase** (<http://ovidsp.ovid.com/ovidweb.cgi?T=JS&PAGE=main&MODE=ovid&D=oemezd>)
**((*"Cushing disease"/ OR *"Cushing syndrome"/ OR "Cushings Disease".ti,ab OR "Cushings Syndrome".ti,ab OR "Cushing's Disease".ti,ab OR "Cushing's Syndrome".ti,ab OR "Cushing Disease".ti,ab OR "Cushing Syndrome".ti,ab OR Cushing*.ti,ab OR "Hypercortisolism".ti,ab OR Hypercortisol*.ti,ab OR "Pituitary ACTH Hypersecretion".ti,ab OR "Inappropriate ACTH Secretion".ti,ab) AND (*"Ketoconazole"/ OR "ketoconazole".ti,ab OR ketoconazol*.ti,ab OR "R-41400".ti,ab OR "R 41400".ti,ab OR "R41,400".ti,ab OR "Nizoral".ti,ab OR *"Metyrapone"/ OR "Metyrapone".ti,ab OR metyrapon*.ti,ab OR "Methopyrapone".ti,ab OR "SU 4885".ti,ab OR "Metopirone".ti,ab OR "Metopiron".ti,ab OR "Métopirone".ti,ab OR *"Mitotane"/ OR** **"Mitotane".ti,ab OR mitotan*.ti,ab OR "o,p-DDD".ti,ab OR "Chlodithane".ti,ab OR "Lysodren".ti,ab OR "Chloditan".ti,ab OR "Khloditan".ti,ab OR *"pasireotide"/ OR "pasireotide".ti,ab OR pasireotid*.ti,ab OR "SOM-230".ti,ab OR "SOM 230".ti,ab OR "SOM230".ti,ab OR *"Mifepristone"/** OR **"Mifepristone".ti,ab OR mifepriston*.ti,ab OR "Mifeprex".ti,ab OR "Mifegyne".ti,ab OR "Mifégyne".ti,ab OR "RU-486".ti,ab OR "RU 486".ti,ab OR "RU486".ti,ab OR "R38486".ti,ab OR "RU-38486".ti,ab OR "RU 38486".ti,ab OR "RU38486".ti,ab OR *"cabergoline"/** OR **"cabergoline".ti,ab OR cabergolin*.ti,ab OR "Galastop".ti,ab OR "FCE 21336".ti,ab OR "FCE-21336".ti,ab OR "Dostinex".ti,ab) AND ("cortisol".ti,ab OR cortisol*.ti,ab OR "Hydrocortisone"/ OR exp "Symptom"/ OR "symptom".ti,ab OR "symptoms".ti,ab OR symptom*.ti,ab OR "Wellbeing"/ OR "well being".ti,ab OR "wellbeing".ti,ab OR exp "Quality of Life"/** OR **"Quality of Life".ti,ab OR "QOL".ti,ab OR exp "Hypertension"/ OR hypertens*.ti,ab OR exp "Diabetes Mellitus"/ OR diabet*.ti,ab)) AND exp "Humans"/ NOT ((exp "case report"/ OR "case report".ti) NOT (exp "Clinical Trial"/ OR "series".ti OR "case study"/ OR exp "review"/ OR "review".ti))**

**Web of Science** (<http://isiknowledge.com/wos>) **ti=("Cushing disease" OR "Cushing syndrome" OR "Cushings Disease" OR "Cushings Syndrome" OR "Cushing's Disease" OR "Cushing's Syndrome" OR "Cushing Disease" OR "Cushing Syndrome" OR Cushing* OR "Hypercortisolism" OR Hypercortisol* OR "Pituitary ACTH Hypersecretion" OR "Inappropriate ACTH Secretion") AND ts=("Ketoconazole" OR "ketoconazole" OR ketoconazol* OR "R-41400" OR "R 41400" OR "R41,400" OR "Nizoral" OR "Metyrapone" OR "Metyrapone" OR metyrapon* OR "Methopyrapone" OR "SU 4885" OR "Metopirone" OR "Metopiron" OR "Métopirone" OR "Mitotane" OR** **"Mitotane" OR mitotan* OR "o,p-DDD" OR "Chlodithane" OR "Lysodren" OR "Chloditan" OR "Khloditan" OR "pasireotide" OR "pasireotide" OR pasireotid* OR "SOM-230" OR "SOM 230" OR "SOM230" OR "Mifepristone"** OR **"Mifepristone" OR mifepriston* OR "Mifeprex" OR "Mifegyne" OR "Mifégyne" OR "RU-486" OR "RU 486" OR "RU486" OR "R38486" OR "RU-38486" OR "RU 38486" OR "RU38486" OR "cabergoline"** OR **"cabergoline" OR cabergolin* OR "Galastop" OR "FCE 21336" OR "FCE-21336" OR "Dostinex") AND ts=("cortisol" OR cortisol* OR "Hydrocortisone" OR "Symptom" OR "symptom" OR "symptoms" OR symptom* OR "Wellbeing" OR "well being" OR "wellbeing" OR "Quality of Life"** OR **"Quality of Life" OR "QOL" OR "Hypertension" OR hypertens* OR "Diabetes Mellitus" OR diabet*) NOT TI=("case report" NOT ("Trial" OR "series" OR "review")) NOT ti=(veterinary OR rabbit OR rabbits OR animal OR animals OR mouse OR mice OR rodent OR rodents OR rat OR rats OR pig OR pigs OR porcine OR horse* OR equine OR cow OR cows OR bovine OR goat OR goats OR sheep OR ovine OR canine OR dog OR dogs OR feline OR cat OR cats)**

**Cochrane** (<http://www.cochranelibrary.com/>)
**(("Cushing disease" OR "Cushing syndrome" OR "Cushings Disease" OR "Cushings Syndrome" OR "Cushing's Disease" OR "Cushing's Syndrome" OR "Cushing Disease" OR "Cushing Syndrome" OR Cushing* OR "Hypercortisolism" OR Hypercortisol* OR "Pituitary ACTH Hypersecretion" OR "Inappropriate ACTH Secretion") AND ("Ketoconazole" OR "ketoconazole" OR ketoconazol* OR "R-41400" OR "R 41400" OR "R41,400" OR "Nizoral" OR "Metyrapone" OR "Metyrapone" OR metyrapon* OR "Methopyrapone" OR "SU 4885" OR "Metopirone" OR "Metopiron" OR "Métopirone" OR "Mitotane" OR** **"Mitotane" OR mitotan* OR "o,p-DDD" OR "Chlodithane" OR "Lysodren" OR "Chloditan" OR "Khloditan" OR "pasireotide" OR "pasireotide" OR pasireotid* OR "SOM-230" OR "SOM 230" OR "SOM230" OR "Mifepristone"** OR **"Mifepristone" OR mifepriston* OR "Mifeprex" OR "Mifegyne" OR "Mifégyne" OR "RU-486" OR "RU 486" OR "RU486" OR "R38486" OR "RU-38486" OR "RU 38486" OR "RU38486" OR "cabergoline"** OR **"cabergoline" OR cabergolin* OR "Galastop" OR "FCE 21336" OR "FCE-21336" OR "Dostinex") AND ("cortisol" OR cortisol* OR "Hydrocortisone" OR "Symptom" OR "symptom" OR "symptoms" OR symptom* OR "Wellbeing" OR "well being" OR "wellbeing" OR "Quality of Life"** OR **"Quality of Life" OR "QOL" OR "Hypertension" OR hypertens* OR "Diabetes Mellitus" OR diabet*)):ti,ab,kw**

**CENTRAL** (<http://archie.cochrane.org/?conferenceabstractredirectTo=http://crso.cochrane.org/login.php&key=4c7b65632dcd2>)
**(("Cushing disease" OR "Cushing syndrome" OR "Cushings Disease" OR "Cushings Syndrome" OR "Cushing's Disease" OR "Cushing's Syndrome" OR "Cushing Disease" OR "Cushing Syndrome" OR Cushing* OR "Hypercortisolism" OR Hypercortisol* OR "Pituitary ACTH Hypersecretion" OR "Inappropriate ACTH Secretion") AND ("Ketoconazole" OR "ketoconazole" OR ketoconazol* OR "R-41400" OR "R 41400" OR "R41,400" OR "Nizoral" OR "Metyrapone" OR "Metyrapone" OR metyrapon* OR "Methopyrapone" OR "SU 4885" OR "Metopirone" OR "Metopiron" OR "Métopirone" OR "Mitotane" OR** **"Mitotane" OR mitotan* OR "o,p-DDD" OR "Chlodithane" OR "Lysodren" OR "Chloditan" OR "Khloditan" OR "pasireotide" OR "pasireotide" OR pasireotid* OR "SOM-230" OR "SOM 230" OR "SOM230" OR "Mifepristone"** OR **"Mifepristone" OR mifepriston* OR "Mifeprex" OR "Mifegyne" OR "Mifégyne" OR "RU-486" OR "RU 486" OR "RU486" OR "R38486" OR "RU-38486" OR "RU 38486" OR "RU38486" OR "cabergoline"** OR **"cabergoline" OR cabergolin* OR "Galastop" OR "FCE 21336" OR "FCE-21336" OR "Dostinex") AND ("cortisol" OR cortisol* OR "Hydrocortisone" OR "Symptom" OR "symptom" OR "symptoms" OR symptom* OR "Wellbeing" OR "well being" OR "wellbeing" OR "Quality of Life"** OR **"Quality of Life" OR "QOL" OR "Hypertension" OR hypertens* OR "Diabetes Mellitus" OR diabet*))**

**Emcare** (<http://ovidsp.ovid.com/ovidweb.cgi?T=JS&NEWS=n&CSC=Y&PAGE=main&D=emcr>)
**((*"Cushing disease"/ OR *"Cushing syndrome"/ OR "Cushings Disease".ti,ab OR "Cushings Syndrome".ti,ab OR "Cushing's Disease".ti,ab OR "Cushing's Syndrome".ti,ab OR "Cushing Disease".ti,ab OR "Cushing Syndrome".ti,ab OR Cushing*.ti,ab OR "Hypercortisolism".ti,ab OR Hypercortisol*.ti,ab OR "Pituitary ACTH Hypersecretion".ti,ab OR "Inappropriate ACTH Secretion".ti,ab) AND ("Ketoconazole"/ OR "ketoconazole".ti,ab OR ketoconazol*.ti,ab OR "R-41400".ti,ab OR "R 41400".ti,ab OR "R41,400".ti,ab OR "Nizoral".ti,ab OR "Metyrapone"/ OR "Metyrapone".ti,ab OR metyrapon*.ti,ab OR "Methopyrapone".ti,ab OR "SU 4885".ti,ab OR "Metopirone".ti,ab OR "Metopiron".ti,ab OR "Métopirone".ti,ab OR "Mitotane"/ OR** **"Mitotane".ti,ab OR mitotan*.ti,ab OR "o,p-DDD".ti,ab OR "Chlodithane".ti,ab OR "Lysodren".ti,ab OR "Chloditan".ti,ab OR "Khloditan".ti,ab OR "pasireotide"/ OR "pasireotide".ti,ab OR pasireotid*.ti,ab OR "SOM-230".ti,ab OR "SOM 230".ti,ab OR "SOM230".ti,ab OR "Mifepristone"/** OR **"Mifepristone".ti,ab OR mifepriston*.ti,ab OR "Mifeprex".ti,ab OR "Mifegyne".ti,ab OR "Mifégyne".ti,ab OR "RU-486".ti,ab OR "RU 486".ti,ab OR "RU486".ti,ab OR "R38486".ti,ab OR "RU-38486".ti,ab OR "RU 38486".ti,ab OR "RU38486".ti,ab OR "cabergoline"/** OR **"cabergoline".ti,ab OR cabergolin*.ti,ab OR "Galastop".ti,ab OR "FCE 21336".ti,ab OR "FCE-21336".ti,ab OR "Dostinex".ti,ab) AND ("cortisol".ti,ab OR cortisol*.ti,ab OR "Hydrocortisone"/ OR exp "Symptom"/ OR "symptom".ti,ab OR "symptoms".ti,ab OR symptom*.ti,ab OR "Wellbeing"/ OR "well being".ti,ab OR "wellbeing".ti,ab OR exp "Quality of Life"/** OR **"Quality of Life".ti,ab OR "QOL".ti,ab OR exp "Hypertension"/ OR hypertens*.ti,ab OR exp "Diabetes Mellitus"/ OR diabet*.ti,ab)) AND exp "Humans"/ NOT ((exp "case report"/ OR "case report".ti) NOT (exp "Clinical Trial"/ OR "series".ti OR "case study"/ OR exp "review"/ OR "review".ti))**

**LWW fulltext** (<http://ovidsp.ovid.com/ovidweb.cgi?T=JS&PAGE=main&MODE=ovidclassic&D=ovft>) **(("Cushing disease" OR "Cushing syndrome" OR "Cushings Disease" OR "Cushings Syndrome" OR "Cushing's Disease" OR "Cushing's Syndrome" OR "Cushing Disease" OR "Cushing Syndrome" OR Cushing* OR "Hypercortisolism" OR Hypercortisol* OR "Pituitary ACTH Hypersecretion" OR "Inappropriate ACTH Secretion").ti AND ("Ketoconazole" OR "ketoconazole" OR ketoconazol* OR "R-41400" OR "R 41400" OR "R41,400" OR "Nizoral" OR "Metyrapone" OR "Metyrapone" OR metyrapon* OR "Methopyrapone" OR "SU 4885" OR "Metopirone" OR "Metopiron" OR "Métopirone" OR "Mitotane" OR** **"Mitotane" OR mitotan* OR "o,p-DDD" OR "Chlodithane" OR "Lysodren" OR "Chloditan" OR "Khloditan" OR "pasireotide" OR "pasireotide" OR pasireotid* OR "SOM-230" OR "SOM 230" OR "SOM230" OR "Mifepristone"** OR **"Mifepristone" OR mifepriston* OR "Mifeprex" OR "Mifegyne" OR "Mifégyne" OR "RU-486" OR "RU 486" OR "RU486" OR "R38486" OR "RU-38486" OR "RU 38486" OR "RU38486" OR "cabergoline"** OR **"cabergoline" OR cabergolin* OR "Galastop" OR "FCE 21336" OR "FCE-21336" OR "Dostinex").ti AND ("cortisol" OR cortisol* OR "Hydrocortisone" OR "Symptom" OR "symptom" OR "symptoms" OR symptom* OR "Wellbeing" OR "well being" OR "wellbeing" OR "Quality of Life"** OR **"Quality of Life" OR "QOL" OR "Hypertension" OR hypertens* OR "Diabetes Mellitus" OR diabet*)).af NOT ("case report" NOT ("Trial" OR "series" OR "review")).ti**

**ScienceDirect fulltext**(<http://www.sciencedirect.com/science?_ob=MiamiSearchURL&_method=requestForm&_temp=all_boolSearch.tmpl&_acct=C000026638&_version=1&_urlVersion=1&_userid=530453&md5=d44bd9fa9076bb9b258a588b309be1e3>) **TITLE("Cushing" OR Cushing* OR "Hypercortisolism" OR Hypercortisol* OR "Pituitary ACTH Hypersecretion" OR "Inappropriate ACTH Secretion") AND TITLE("ketoconazole" OR ketoconazol* OR "R-41400" OR "R 41400" OR "Nizoral" OR "Metyrapone" OR metyrapon* OR "Methopyrapone" OR "SU 4885" OR "Metopirone" OR "Metopiron" OR "Métopirone" OR "Mitotane" OR** **"Mitotane" OR mitotan* OR "Chlodithane" OR "Lysodren" OR "Chloditan" OR "Khloditan" OR "pasireotide" OR pasireotid* OR "SOM-230" OR "SOM 230" OR "SOM230" OR "Mifepristone" OR mifepriston* OR "Mifeprex" OR "Mifegyne" OR "Mifégyne" OR "RU-486" OR "RU 486" OR "RU486" OR "R38486" OR "RU-38486" OR "RU 38486" OR "RU38486" OR "cabergoline" OR cabergolin* OR "Galastop" OR "FCE 21336" OR "FCE-21336" OR "Dostinex") AND (cortisol* OR "Hydrocortisone" OR "Symptom" OR "symptom" OR "symptoms" OR symptom* OR "Wellbeing" OR "well being" OR "wellbeing" OR "Quality of Life"** OR **"Quality of Life" OR "QOL" OR "Hypertension" OR hypertens* OR "Diabetes Mellitus" OR diabet*) NOT TITLE("case report")**
