# Supplementary material for: Effectiveness of medical treatment for Cushing’s syndrome: a systematic review and meta-analysis
Source: Pituitary. 2018 May 31;21(6):631–41. doi: 10.1007/s11102-018-0897-z (PMC6244780; doi:10.1007/s11102-018-0897-z)
Supplement: Supplementary file 2 — Online Resource 2 Study characteristics (DOCX 33 KB) [file 11102_2018_897_MOESM2_ESM.docx]

**Pituitary**

**Effectiveness of medical treatment for Cushing’s syndrome – a systematic review and meta-analysis.**

Leonie H.A. Broersen^1,2,3^, Meghna Jha^3^, Nienke R. Biermasz^1,2^, Alberto M. Pereira^1,2^, Olaf M. Dekkers^1,2,4^

^1^Department of Medicine, division of Endocrinology, Leiden University Medical Centre, Albinusdreef 2, 2333 ZA, Leiden, The Netherlands
^2^Center for Endocrine Tumors Leiden (CETL), Leiden University Medical Center, Albinusdreef 2, 2333 ZA, Leiden, The Netherlands  ^3^Department of Endocrinology, Diabetes and Nutrition, Charité Universitätsmedizin Berlin, Chariteplatz 1, 10117, Berlin, Germany

^4^Department of Clinical Epidemiology, Leiden University Medical Center, Albinusdreef 2, 2333 ZA, Leiden, The Netherlands

Corresponding author: L.H.A. Broersen, L.H.A.Broersen@lumc.nl, +31 (0)71-5263082

Online Resource 2: study characteristics.

|  | **Study period** | **Center (city, country)** | **Study type** | **Type of Cushing syndrome** | **Medical treatment (mean dose (range))** | **Treatment duration, mean (range)** | **Age, mean (range) in years** | **Gender, female/male** | **Duration of follow-up, mean (range) in years** |
| --- | --- | --- | --- | --- | --- | --- | --- | --- | --- |
| *Mitotane* |  |  |  |  |  |  |  |  |  |
| Baudry 2012 | 1993-2009 | Paris, France | Cohort | Pituitary | Mitotane (1^st^ line: 2.5 (1.1-4.3) g/d / 2^nd^ line: 2.4 (0.9-6.1) g/d) | 1^st^ line: 6.9 (0.3-114.9)^a^ / 2^nd^ line: 16.4 (0.8-68.9)^a^ months | 1^st^ line: 39 (14-71) / 2^nd^ line: 34 (14-61)^b^ | 59/17^b^ | 5.9 (2.4-10.5)^b^ |
| Luton 1979 | Not reported | Paris, France | Cohort | Pituitary | Mitotane (4-12 g/d); part also radiotherapy | 90-1020 days | 41 (13-72) | 49/13 | 0.2-6.7 |
| Maher 1992 | 1982-1992 | London, United Kingdom | Cohort | Mixed etiology (pituitary, adrenal tumor (unclear if this means adenoma or carcinoma), ectopic) | Mitotane (0.5-6.0 g/d) | 2-77 months | Not reported | 22/8 | Not reported |
| Orth 1971 | 1954-1971 | Nashville, TN, USA | Cohort | Mixed etiology (pituitary, adrenal carcinoma) | Mitotane (pituitary: 3-6 g/d; adrenal: not reported) | Pituitary: 4-6 months; adrenal: not reported | Pituitary: 42 (22-68); adrenal: 53.7 (32-68) | Pituitary 7/1; adrenal: 3/0 | Pituitary: 3 (1-5); adrenal: 1.3 (0.8-2.0) |
| Schteingart 1980 | Not reported | Ann Arbor, MI, USA | Cohort | Pituitary | Mitotane (1.2 (0.5 twice per week-4.0 g/d) g/d); 33 patients also radiotherapy, 3 bilateral adrenalectomy | 6-89 months | Not reported | 24/12 | Not reported |
| *Pasireotide* |  |  |  |  |  |  |  |  |  |
| Boscaro 2009 | Not reported | 10 centers in 5 countries | Trial (single-arm) | Pituitary | Pasireotide (1200 µg/d) | 15 days | 40.4 (22-73) | 21/8 | 0.04 |
| Colao 2012 | Not reported | Multiple centers | Randomized trial (both treatment arms: pasireotide 600 µg vs. pasireotide 900 µg) | Pituitary | Pasireotide (mean 1165-1875 µg/d depending on treatment arm and time after start treatment) | 10.8 (0.03-37.8) months | 40 (18-71) | 126/36 | Not reported |
| *Cabergoline* |  |  |  |  |  |  |  |  |  |
| Burman 2016 | Not reported | 4 centers in Sweden | Trial (single-arm) | Pituitary | Cabergoline (median 5 (2.5-5) mg/wk) | 6 weeks | 44.6 (20-66) | 19/1 | 0.1 |
| Godbout 2010 | 2002-2006 | Buenos Aires, Argentina and Montréal, Canada | Cohort | Pituitary | Cabergoline (1.5 (0.5-4.0) mg/wk) | Not reported | 20-67 | 25/5 | Not reported |
| Pivonello 2009 | Not reported | Naples, Italy | Trial (single-arm) | Pituitary | Cabergoline (1-7 mg/wk) | 3-24 months | 24-60 | 15/5 | 0.3-2-0 |
| *Ketoconazole* |  |  |  |  |  |  |  |  |  |
| Castinetti 2014 | 1995-2012 | 14 centers in France | Cohort | Pituitary | Ketoconazole (mean 668-780 mg/d depending on control status (200-1200)) | Mean 9.7-27.6 months depending on control status (0.03-135) | 41.9 (8-87) | 156/44 | Not reported |
| Fallo 1993 | 1987-1993 | Padua, Italy | Cohort | Mixed etiology (pituitary, adrenal adenoma and carcinoma, ectopic) | Ketoconazole (400-800 mg/d); part also antihypertensive | 4-30 months | Ketoconazole only: 39 (SEM 2); ketoconazole and antihypertensive: 38 (SEM 3) | 19/5 | Maximum 6 |
| Ghervan 2015 | 2010-2013 | Cluj-Napoca, Romania | Cohort | Mixed etiology (pituitary, adrenal hyperplasia, ectopic)^c^ | Ketoconazole (591.7 (300-800 mg/d)) | 15.5 (1-75) months | 35.9 (4-63) | 7/5 | Controlled 0.6; uncontrolled 2.5 |
| Luisetto 2001 | 1990-2000 | Padua, Italy | Cohort | Pituitary | Ketoconazole (300-600 mg/d) | 44.9 (10-100) months | 45.1 (SD 7.1) | 9/1 | Normalized cortisol 4.8 (SD 3.9); not normalized cortisol 3.6 (SD 2.3) |
| Moncet 2007 | Not reported | Buenos Aires, Argentina | Cohort | Mixed etiology (pituitary, adrenal adenoma and carcinoma, ectopic) | Ketoconazole (600 (200-1200) mg/d) | 0.8 (0.04-13) years | 38 (14-63) | 44/10 | Not reported |
| Sonino 1991 | 1985-1991 | Padua, Italy | Cohort | Mixed etiology (pituitary, adrenal adenoma, hyperplasia, and carcinoma, ectopic) | Ketoconazole (576.5 (400-1200 mg/d)); part also radiotherapy | 7.5 months (4 days – 3 years) | 38.5 (14-67) | 28/6 | Not reported |
| Stiefel 2002 | Not reported | Seville, Spain | Trial (single-arm) | Mixed etiology (pituitary, adrenal adenoma and hyperplasia) | Ketoconazole (800 mg/d) | 1 week | 32.2 (SD 10.4) | 13/2 | Not reported |
| Winquist 1995 | 1980-1992 | Toronto, Canada | Cohort | Ectopic | Ketoconazole (400-1200 mg/d) | 26 (3-1059) days^a^ | 59 (44-84)^a^ | 4/11 | Not reported |
| *Metyrapone* |  |  |  |  |  |  |  |  |  |
| Child 1976 | Not reported | London, United Kingdom | Trial (single-arm) | Mixed etiology (pituitary, adrenal adenoma, ectopic) | Metyrapone (2 g/d); all patients also amino-glutethimide | 2 weeks – 1 year | Not reported | 15/3 | Not reported |
| Jeffcoate 1977 | Not reported | London, United Kingdom | Cohort | Pituitary | Metyrapone (0.5-4.0 g/d); part also radiotherapy | 22 (2-66) months | 41 (16-60) | 11/2 | Not reported |
| Jeffcoate 1979 | 1968-1978 (partially double population with Jeffcoate 1977) | London, United Kingdom | Cohort | Mixed etiology (pituitary, adrenal adenoma and carcinoma (separately described), ectopic)^c^ | Metyrapone (0.5-6.0 g/d); part also radiotherapy | Not reported | Mixed group without adrenal carcinoma: 43 (15-66); adrenal carcinoma: 46 (44-48) | Mixed group without adrenal carcinoma: 15/4; adrenal carcinoma: 1/1 | Not reported |
| Ross 1979 | 1971-1975 | Newcastle upon Tyne, United Kingdom | Trial (single-arm) | Pituitary | Metyrapone (dose not reported); all patients also radiotherapy, part also amino-glutethimide | 2 years | 39.5 (25-66) | 7/3 | Not reported |
| Verhelst 1991 | Not reported | London, United Kingdom | Trial (single-arm) | Mixed etiology (pituitary, adrenal adenoma and carcinoma, ectopic)^d^ | Metyrapone (pituitary: 2.25 (0.5-6.0); adrenal 1.75 (0.75-6.0); ectopic 4.0 (1.0-6.0) g/d)^a^; part also radiotherapy | Pituitary: 1 week – 140 months; adrenal: 2-8 weeks; ectopic: 1 week – 44 months | Pituitary: (14-68); adrenal: (9-68); ectopic: (33-73) | Pituitary: 43/14; adrenal: 12/4; ectopic: 10/8 | Not reported |
| *Mifepristone* |  |  |  |  |  |  |  |  |  |
| Castinetti 2009 | Not reported | 7 European centers (France, Germany, Italy, Spain) | Cohort | Mixed etiology (pituitary, adrenal hyperplasia and carcinoma, ectopic) | Mifepristone (600 (400-2000 mg/d)^a^ | 2.5 (0.2-24) months^a^ | 48.2 (20-64) | 13/7 | Not reported |
| Fleseriu 2012 | 2008-2011 | 17 centers in the USA | Trial (single-arm) | Mixed etiology (pituitary, adrenal carcinoma, ectopic) | Mifepristone (732 (300-1200) mg/d) | Maximum 24 weeks | 45.4 (26-71) | 35/15 | 0.6 |
| *Multiple medical agents* |  |  |  |  |  |  |  |  |  |
| Barbot 2014 | Not reported | Padua, Italy | Randomized trial (both treatment arms: first cabergoline, then add ketoconazole vs. first ketoconazole, then add cabergoline) | Pituitary | Cabergoline and/or ketoconazole  (C: 0.5-3.0 mg/wk; K: 200-600 mg/d) | 12-18 months | 52 (33-70)^a^ | 12/2 | 1.0-1.5 |
| Corcuff 2015 | Not reported | Le Kremlin-Bicêtre and Pessac, France | Cohort | Mixed etiology (adrenal carcinoma, ectopic) | Metyrapone and ketoconazole (part also mitotane) (M: median 1750-2875 mg/d; K: median 800-1000 mg/d) | 1 month | Ectopic: 53 (24-71)^a^; adrenal 60 (20-71)^a^ | 8/14 | 0.1-4 |
| Daniel 2015 | 1997-2013 | 13 university centers in England and Wales | Cohort | Mixed etiology (pituitary, adrenal adenoma, hyperplasia, and carcinoma, ectopic) | Metyrapone (part combined with other medication, most often ketoconazole or mitotane) (500-4000 mg/d) | 8 months (3 days – 11.6 years) | 49.6 (1-81) | 138/57 | Not reported |
| Donadille 2010 | 1990-2006 | Paris, France | Cohort | Ectopic | Mitotane (part also amino-glutethimide, ketoconazole, or metyrapone) (3.3 (1.5-6.0) g/d) | 1.8 (0.06-9.7) years | 53.7 (32-80) | 5/18 | 8.0 (0.3-34) |
| Feelders 2010 | Not reported | 4 centers in the Netherlands | Trial (single-arm) | Pituitary | Pasireotide (300-750 µg/d), add cabergoline (0.5-1.5 mg/48 h), add ketoconazole (600 µg/d) according to protocol | 80 days | 45.7 | 13/4 | 0.2 |
| Ferriere 2017 | 2003-2015 | 13 centers in France and Belgium | Cohort | Pituitary | Cabergoline (only treatment: 2.3 (0.5-6.0)^a^ mg/wk; add-on therapy: 1.0 (0.5-3.5)^a^ mg/wk); part also ketoconazole or metyrapone. | Only treatment: 7 (1-105)^a^ months; add-on therapy: 19 (1-240)^a^ months | 37.5 (7-78) | 50/12 | Not reported |
| Valassi 2012 | 1983-2010 | Barcelona, Spain | Cohort | Mixed etiology (pituitary, adrenal adenoma, ectopic) | Ketoconazole and/or metyrapone (K: 200-1000 mg/d, M: 0.75-4.5 g/d) | 4 (1-30.7)^a^ months | 41 (7-70) | 54/8 | 9.0 (0.3-23.0)^a^ |
| Van den Bosch 2014 | 1990-2010 | Utrecht, the Netherlands | Cohort | Pituitary | Ketoconazole and/or metyrapone (K: 720 mg/d, M: 2.5 g/d) | 174 (123-261)^e^ days | 50 (16-65) | 25/8 | 11.5 (0.2-22.5)^a^ |
| Van der Pas 2013 | Not reported (double population with Feelders 2010) | 4 centers in the Netherlands | Trial (single-arm) | Pituitary | Pasireotide (750 µg/d), add cabergoline (0.5-1.5 mg/48 h), add ketoconazole (600 µg/d) according to protocol | 80 days | 45.5 (22-67) | 12/4 | 0.2-1.0 |
| Vilar 2010 | Not reported | Recife, Brazil | Trial (single-arm) | Pituitary | Cabergoline (1-3 mg/wk), part also ketoconazole (100-400 mg/d) | 6-12 months | 42.8 (34-52) | 8/4 | Not reported |

^a^Median + range
^b^Includes patients lost to follow-up
^c^Also included in separate analyses on pituitary Cushing disease
^d^Also included in separate analyses on pituitary Cushing disease and ectopic Cushing syndrome ^e^Median + interquartile range
