# Supplementary material for: Effectiveness of medical treatment for Cushing’s syndrome: a systematic review and meta-analysis
Source: Pituitary. 2018 May 31;21(6):631–41. doi: 10.1007/s11102-018-0897-z (PMC6244780; doi:10.1007/s11102-018-0897-z)
Supplement: Supplementary file 3 — Online Resource 3 Risk of bias assessment (DOCX 28 KB) [file 11102_2018_897_MOESM3_ESM.docx]

**Pituitary**

**Effectiveness of medical treatment for Cushing’s syndrome – a systematic review and meta-analysis.**

Leonie H.A. Broersen^1,2,3^, Meghna Jha^3^, Nienke R. Biermasz^1,2^, Alberto M. Pereira^1,2^, Olaf M. Dekkers^1,2,4^

^1^Department of Medicine, division of Endocrinology, Leiden University Medical Centre, Albinusdreef 2, 2333 ZA, Leiden, The Netherlands
^2^Center for Endocrine Tumors Leiden (CETL), Leiden University Medical Center, Albinusdreef 2, 2333 ZA, Leiden, The Netherlands  ^3^Department of Endocrinology, Diabetes and Nutrition, Charité Universitätsmedizin Berlin, Chariteplatz 1, 10117, Berlin, Germany

^4^Department of Clinical Epidemiology, Leiden University Medical Center, Albinusdreef 2, 2333 ZA, Leiden, The Netherlands

Corresponding author: L.H.A. Broersen, L.H.A.Broersen@lumc.nl, +31 (0)71-5263082

Online Resource 3: risk of bias assessment.

|  | **Loss to follow-up, N (%)** | **Inclusion of patients** | **Criteria for diagnosis of Cushing’s syndrome** | **Outcome measurement for cortisol normalization** | **Reporting of outcome definition** | **Description of protocol for laboratory measurements** | **Description of dose and duration of intervention** |
| --- | --- | --- | --- | --- | --- | --- | --- |
| *Mitotane* |  |  |  |  |  |  |  |
| Baudry 2012 | 9 (10.6%) not in analyses, short-term treatment before surgery | Consecutive | Mentioned | Urinary free cortisol, morning plasma cortisol & after 250 µg ACTH | Adequate | Adequate | Adequate |
| Luton 1979 | Not reported | Not reported | Mentioned | Urinary free cortisol, plasma cortisol, 17-hydroxycorticosteroids | Adequate | Not adequate | Not adequate |
| Maher 1992 | Not reported | Not reported | Not mentioned | Not relevant | Not adequate | Adequate | Not adequate |
| Orth 1971 | Not reported | Consecutive | Mentioned | Urinary 17-OHCS, plasma cortisol | Adequate | Not adequate | Not adequate |
| Schteingart 1980 | Not reported | Not reported | Mentioned | Urinary free cortisol, plasma cortisol day curve, cortisol secretion rate | Not adequate | Adequate | Not adequate |
| *Pasireotide* |  |  |  |  |  |  |  |
| Boscaro 2009 | 0 (0%) | Not reported | Mentioned | Urinary free cortisol | Adequate | Adequate | Adequate |
| Colao 2012 | Not reported | 162 of 329 screened patients included | Mentioned | Urinary free cortisol | Adequate | Not adequate | Adequate |
| *Cabergoline* |  |  |  |  |  |  |  |
| Burman 2016 | 0 (0%) | Not reported | Mentioned | Urinary free cortisol | Adequate | Adequate | Adequate |
| Godbout 2010 | Not reported | Consecutive | Mentioned | Urinary free cortisol | Adequate | Adequate | Not adequate |
| Pivonello 2009 | 12 (60%) lost per protocol after stop treatment because of resistance or intolerance | Not reported | Mentioned | Urinary free cortisol | Adequate | Adequate | Adequate |
| *Ketoconazole* |  |  |  |  |  |  |  |
| Castinetti 2014 | 3 (1.5%) | Not reported | Mentioned | Urinary free cortisol | Adequate | Not adequate | Adequate |
| Fallo 1993 | Not reported | Consecutive patients with Cushing and hypertension | Mentioned | Urinary free cortisol | Not adequate | Not adequate | Not adequate |
| Ghervan 2015 | Not reported | Consecutive | Mentioned | Morning serum cortisol | Adequate | Not adequate | Adequate |
| Luisetto 2001 | Not reported | Not reported | Mentioned | Urinary free cortisol | Adequate | Adequate | Not adequate |
| Moncet 2007 | 2 (3.7%) | Not reported | Mentioned | Urinary free cortisol | Adequate | Not adequate | Adequate |
| Sonino 1991 | Not reported | Consecutive | Mentioned | Urinary free cortisol, morning plasma cortisol | Not adequate | Adequate | Adequate |
| Stiefel 2002 | Not reported | Not reported | Mentioned | Urinary free cortisol | Not adequate | Adequate | Adequate |
| Winquist 1995 | Not reported | Consecutive | Mentioned | Urinary free cortisol, plasma cortisol | Adequate | Not adequate | Adequate |
| *Metyrapone* |  |  |  |  |  |  |  |
| Child 1976 | Not reported | Consecutive | Mentioned | Urinary free cortisol, morning plasma cortisol | Not adequate | Adequate | Adequate |
| Jeffcoate 1977 | Not reported | Consecutive | Mentioned | Plasma cortisol day curve | Adequate | Adequate | Not adequate |
| Jeffcoate 1979 | Not reported | Consecutive patients with psychiatric examination | Mentioned | Not relevant | Not adequate | Not relevant | Not adequate |
| Ross 1979 | 1 (7%) | Consecutive | Mentioned | Urinary free cortisol, plasma cortisol | Not adequate | Adequate | Not adequate |
| Verhelst 1991 | Not reported | Consecutive | Mentioned | Serum cortisol | Adequate | Adequate | Not adequate |
| *Mifepristone* |  |  |  |  |  |  |  |
| Castinetti 2009 | Not reported | Not reported | Mentioned | Not relevant | Not adequate | Not adequate | Adequate |
| Fleseriu 2012 | 7 (14%) | Consecutive patients with diabetes mellitus/impaired glucose tolerance/hypertension | Mentioned | Urinary free cortisol, late night salivary cortisol, serum cortisol | Adequate | Adequate | Adequate |
| *Multiple medical agents* |  |  |  |  |  |  |  |
| Barbot 2014 | 0 (0%) in first 12 months | Not reported | Mentioned | Urinary free cortisol, late night salivary cortisol | Adequate | Adequate | Adequate |
| Corcuff 2015 | Not reported | Not reported | Mentioned | Urinary free cortisol, midnight plasma cortisol | Adequate | Adequate | Adequate |
| Daniel 2015 | 2 (1%) | Consecutive | Not mentioned | Urinary free cortisol, morning serum cortisol and day curve | Adequate | Adequate | Adequate |
| Donadille 2010 | Not reported | Consecutive | Mentioned | Urinary free cortisol, morning plasma cortisol, midnight salivary cortisol | Not adequate | Adequate | Adequate |
| Feelders 2010 | Not reported | Not reported | Not mentioned | Urinary free cortisol | Adequate | Not adequate | Adequate |
| Ferriere 2017 | Not reported | Consecutive patients with ≥1 month of cabergoline use | Mentioned | Urinary free cortisol | Adequate | Adequate | Adequate |
| Valassi 2012 | 41 (40%) before start study | 62 of 103 eligible Cushing’s syndrome patients included | Mentioned | Urinary free cortisol | Adequate | Adequate | Not adequate |
| Van den Bosch 2014 | 3 (8%) excluded after stop treatment | Consecutive patients with pretreatment for surgery | Mentioned | Urinary free cortisol, serum cortisol day curve | Adequate | Adequate | Adequate |
| Van der Pas 2013 | Not reported | Not reported | Not mentioned | Urinary free cortisol, plasma cortisol, salivary cortisol | Adequate | Not relevant | Adequate |
| Vilar 2010 | Not reported | Selection without mentioned criteria | Mentioned | Urinary free cortisol | Adequate | Adequate | Adequate |
