# Supplementary material for: Effectiveness of medical treatment for Cushing’s syndrome: a systematic review and meta-analysis
Source: Pituitary. 2018 May 31;21(6):631–41. doi: 10.1007/s11102-018-0897-z (PMC6244780; doi:10.1007/s11102-018-0897-z)
Supplement: Supplementary file 4 — Online Resource 4 Study outcomes (DOCX 33 KB) [file 11102_2018_897_MOESM4_ESM.docx]

**Pituitary**

**Effectiveness of medical treatment for Cushing’s syndrome – a systematic review and meta-analysis.**

Leonie H.A. Broersen^1,2,3^, Meghna Jha^3^, Nienke R. Biermasz^1,2^, Alberto M. Pereira^1,2^, Olaf M. Dekkers^1,2,4^

^1^Department of Medicine, division of Endocrinology, Leiden University Medical Centre, Albinusdreef 2, 2333 ZA, Leiden, The Netherlands
^2^Center for Endocrine Tumors Leiden (CETL), Leiden University Medical Center, Albinusdreef 2, 2333 ZA, Leiden, The Netherlands  ^3^Department of Endocrinology, Diabetes and Nutrition, Charité Universitätsmedizin Berlin, Chariteplatz 1, 10117, Berlin, Germany

^4^Department of Clinical Epidemiology, Leiden University Medical Center, Albinusdreef 2, 2333 ZA, Leiden, The Netherlands

Corresponding author: L.H.A. Broersen, L.H.A.Broersen@lumc.nl, +31 (0)71-5263082

Online Resource 4: study outcomes.

|  | **Total number of patients (Number pre-treated before surgery)** | **Normalization of cortisol (N)** | **Clinical improvement** | **Well-being / Quality of life** | **Side effects** | **Number of patients with primary treatment** | **Normalization cortisol (N) / Clinical improvement** | **Number of patients with secondary treatment** | **Normalization cortisol (N) / Clinical improvement** |
| --- | --- | --- | --- | --- | --- | --- | --- | --- | --- |
| *Mitotane* |  |  |  |  |  |  |  |  |  |
| Baudry 2012 | 67 (9) | 48 | Significant improvement metabolic parameters (BMI, diastolic blood pressure, glucose, cholesterol) | Not reported | 19x stop therapy due to intolerance | 49 | Not reported | 27 | Not reported |
| Luton 1979 | 62 | 54 | Not reported | Not reported | 38x digestive discomfort, 1x rash, 1x chloasma, 8x gynecomastia | Not reported | Not reported | Not reported | Not reported |
| Maher 1992 | 30 | Not reported | Not reported | Not reported | In all 21 tested patients mean increase cholesterol 68% | Not reported | Not reported | Not reported | Not reported |
| Orth 1971 | Pituitary: 8; adrenal: 3 | Pituitary: 8; adrenal: 0 | Not reported | Not reported | Pituitary: 4x nausea; adrenal: 3x nausea and ataxia | Not reported | Not reported | Not reported | Not reported |
| Schteingart 1980 | 36 (0) | 29 | Not reported | Not reported | 32x anorexia / nausea, 18x decreased memory, 6x gynecomastia | Not reported | Not reported | Not reported | Not reported |
| *Pasireotide* |  |  |  |  |  |  |  |  |  |
| Boscaro 2009 | 39 (safety population); 29 (efficacy population) | 5 | Not reported | Not reported | 3x serious adverse event (2x severe hyperglycemia, of which 1x stop therapy, 1x cardiac related events in known cardiac disease), 31x mild adverse event (3x dose reduction) | Not reported | Not reported | Not reported | Not reported |
| Colao 2012 | 162 (0) | 50 (out of 103 tested) | Mean difference blood pressure  -6.1 mmHg, triglycerides  -2 mg/dL, LDL  -15 mg/dL, weight -6.7 kg | Mean difference health-related quality of life 11.1 points (CushingQoL questionnaire) | 26 patients stopped therapy due to adverse events. Various side effects are seen in up to 58% of patients per side effect. | Not reported | Not reported | Not reported | Not reported |
| *Cabergoline* |  |  |  |  |  |  |  |  |  |
| Burman 2016 | 20 (18) | 2 | No improvement in weight, blood pressure, glucose or HbA1c | Not reported | 3x dose reduction, 10x side effects without dose reduction | 19 | Not reported | 1 | Not reported |
| Godbout 2010 | 30 | 11 | 11x improvement clinical signs | Not reported | 3x dizziness and nausea, without therapy withdrawal | 3 | 1 /  Not reported | 27 | 10 /  Not reported |
| Pivonello 2009 | 20 (0) | 13 | 5x obesity (N=7), 4x hypertension (N=4), 1x diabetes mellitus (N=2), 1x glucose intolerance (N=3)^a^ | Not reported | 2x stop therapy due to asthenia, 4x mild asthenia, 1x dizziness, without stop therapy | 0 | Not applicable | 20 | 10 /  5x obesity (N=7), 4x hypertension (N=4), 1x diabetes mellitus (N=2), 1x glucose intolerance (N=3) |
| *Ketoconazole* |  |  |  |  |  |  |  |  |  |
| Castinetti 2014 | 200 (40) | 97 | 88x clinical signs, 49x hypertension, 15x hypokalemia, 31x diabetes mellitus | Not reported | 41x stop therapy due to intolerance | 72 | 35 /  35x clinical signs, 21x hypertension, 7x hypokalemia, 15x diabetes mellitus | 128 | 62 / 53x clinical signs, 28x hypertension, 8x hypokalemia, 16x diabetes mellitus |
| Fallo 1993 | 24 (24) | 23 | 22x hypertension | Not reported | 1x increased liver enzymes, 1x dyspepsia | Not reported | Not reported | Not reported | Not reported |
| Ghervan 2015 | 12 (4) Pituitary: 10 Adrenal: 1 Ectopic: 1 | 8 Pituitary: 6 Adrenal: 1 Ectopic: 1 | 7x cholesterol (N=12), 6x hypertension (N=8), 3x glucose tolerance (N=3), 5x weight (N=10)^a^ | Not reported | 1x dose reduction due to hypoadrenalism | 8 | Not reported | 4 | Not reported |
| Luisetto 2001 | 10 (0) | 5 | Not reported | Not reported | Not reported | 0 | Not reported | 10 | Not reported |
| Moncet 2007 | 54 (27) | 44 | 33x hypertension (N=41), 20x myopathy (N=27), 23x obesity (N=45), 10x edema (N=20), 21x menstrual disturbance (N=35), 8x diabetes mellitus (N=11)^a^ | Not reported | 18 patients side effects, treatment withdrawal in 4 (transient subclinical hepatic injury 6x, adrenal failure 10x, rash 3x, digestive intolerance 2x) | 38 | Not reported | 16 | Not reported |
| Sonino 1991 | 34 | 30 | 20x hypertension (N=21), 6x hyperglycemia (N=6), 6x hypokalemia (N=8), 5x hirsutism, 2x amenorrhea, 23x cholesterol^a^ | Not reported | 1x rash, 1x symptomatic liver toxicity (treatment withdrawal), 3x asymptomatic lever enzyme increase, 4x gastrointestinal side effect, 1x gynecomastia | Not reported | Not reported | Not reported | Not reported |
| Stiefel 2002 | 15 | Not reported | Mean difference systolic blood pressure -35.6 mmHg, diastolic blood pressure  -30.4 mmHg, mean blood pressure -32.0 mmHg | Not reported | Not reported | Not reported | Not reported | Not reported | Not reported |
| Winquist 1995 | 15 | 7 | 13x hypokalemia (N=14), 8x metabolic alkalosis (N=11), 7x diabetes mellitus (N=10), 3x edema (N=8), 3x muscle weakness, 2x cushingoid features (N=7), 8x hypertension (N=8)^a^ | Not reported | 4x hypoadrenalism (2x interruption therapy) | Not reported | Not reported | Not reported | Not reported |
| *Metyrapone* |  |  |  |  |  |  |  |  |  |
| Child 1976 | 18 | 15 | 3x severe myopathy (N=3), 6x diabetes mellitus (N=6), 11x general improvement (N=18)^a^ | Not reported | 14 patients side effects: 5x rash, 4x drowsiness, 1x nausea and dizziness, 1x slurring of speech and ataxia, 3x drowsiness and/or dizziness (8x change of treatment) | Not reported | Not reported | Not reported | Not reported |
| Jeffcoate 1977 | 13 | Not reported | 5x glucose intolerance (N=7), 7x hypertension (N=12)^a^ | Not reported | 5x hirsutism / persisting acne (1x stop therapy) | Not reported | Not reported | Not reported | Not reported |
| Jeffcoate 1979 | Mixed group without adrenal carcinoma: 19; adrenal carcinoma: 2 | Not reported | Mixed group without adrenal carcinoma: 11x psychiatric symptoms; adrenal carcinoma: 1x psychiatric symptoms | Not reported | Not reported | Not reported | Not reported | Not reported | Not reported |
| Ross 1979 | 10 | 6 | Not reported | Not reported | Not reported | Not reported | Not reported | Not reported | Not reported |
| Verhelst 1991 | Pituitary: 57 (47); adrenal: 16 (12); ectopic: 18 (9) | Not reported | Mixed group (no separate data): 89% bloated face, 79% muscle weakness, 73% psychiatric disturbance, 9% hirsutism, 73% hypertension, 82% glucose intolerance/ diabetes mellitus (assessed in 75 patients) | Not reported | Mixed group (no separate data): 10x transient hypoadrenalism (dose reduction), 5x transient mild hypokalemia, 1x dangerous hypokalemia, 6x edema, 3x rash, 12x dizziness, 3x hirsutism/acne (assessed in 75 patients) | Pituitary: 53; adrenal and ectopic: not reported | Pituitary: 40 / Not reported; adrenal and ectopic: not reported | Pituitary: 24; adrenal and ectopic: not reported | Pituitary: 20 / Not reported; adrenal and ectopic: not reported |
| *Mifepristone* |  |  |  |  |  |  |  |  |  |
| Castinetti 2009 | 20 (1) | Not reported | 15x improved clinical signs (N=20), 3x psychiatric symptoms (N=4), 4x blood pressure (N=9), 4x diabetes mellitus (N=7)^a^ | Not reported | 11x hypokalemia (3x stop treatment), 3x hypertension, 4x increased cortisol, 3x adrenal insufficiency (dose reduction), 2x fatigue, 1x hypoglycemia | 2 | Not reported | 18 | Not reported |
| Fleseriu 2012 | 50 | Not reported | 15x glucose (N=25), 17x hypertension (N=40), overall clinical improvement 87% measured in 46 patients, weight  -5.7%, waist -6.8 cm in women,  -8.4 cm in men, percent total body fat -3.6%^a^ | SF-36: mental +5.4 points and physical +4.2 points | 12x hypertension, 31x increased ACTH and cortisol (N=43), 2x adrenal insufficiency; in total 44 patients adverse event; 7x discontinued treatment, 20x interruptions/dose reduction | Not reported | Not reported | Not reported | Not reported |
| *Multiple medical agents* |  |  |  |  |  |  |  |  |  |
| Barbot 2014 | 14 | 11 | Most patients show partial regression of symptoms (waist, BMI, less antihypertensives, better glucose control) | Not reported | 2x dose reduction (1x high transaminases, 1x skin rash) | Not reported | Not reported | Not reported | Not reported |
| Corcuff 2015 | 22 | 15 | Ectopic/adrenal: 82%/37% hypokalemia, 25%/38% stop antihypertensives, 28%/0% stop antidiabetics, mean difference systolic blood pressure -45/-30 mmHg, diastolic blood pressure  -15/-20 mmHg, fasting glucose  -6.6/-1.1 mmol/L | Not reported | 2x increased liver enzymes (stop treatment), 11x nausea | Not reported | Not reported | Not reported | Not reported |
| Daniel 2015 | 195 (124) | Not reported | Not reported | Not reported | 48 patients had 57 events: gastrointestinal upset 23%, hypoadrenalism 7%; 15% dose reduction, 23% withdrawal (temporarily or permanent) | 164 | Not reported | 31 | Not reported |
| Donadille 2010 | 23 | 21 | 12x hypertension (N=19), 4x diabetes mellitus (N=10)^a^ | Not reported | 1x stop treatment due to severe increase liver enzymes, 20x adrenal insufficiency, 15x digestive signs, 6x neurological signs, 1x transient gynecomastia, 12x hyper-cholesterolemia, 1x coronary event | Not reported | Not reported | Not reported | Not reported |
| Feelders 2010 | 17 | 15 | Mean difference systolic blood pressure -12 mmHg, diastolic blood pressure  -8 mmHg, waist  -4.2 cm, weight  -2.4 kg | Not reported | 9x disturbance in glucose homeostasis | Not reported | Not reported | Not reported | Not reported |
| Ferriere 2017 | 62 (5) | 28 | In complete responders: 7x weight, 11x glucose control, 9x hypertension | Not reported | 8x stop therapy due to poor tolerance, 9x adverse events without stop therapy (assessed in 57 patients) | 9 | Not reported | 53 | Not reported |
| Valassi 2012 | 62 (60) | 32 | 20x complete control of nine symptoms, and hypertension and diabetes mellitus | Not reported | Various side effects are seen in 1-30 patients per side effect. | 62 | 32 /  20x complete control of nine symptoms, and hypertension and diabetes mellitus | 0 | Not applicable |
| Van den Bosch 2014 | 33 (33) | 16 | Not reported | Not reported | 1x stop therapy due to side effects, 17x adjust therapy (of which 3x hepatotoxicity) | 33 | 16 /  Not reported | 0 | Not applicable |
| Van der Pas 2013 | 16 | Double data | Not reported | Quality of life: emotional reaction improved (NHP), more pain after study period (RAND 36) | Not reported | Not reported | Not reported | Not reported | Not reported |
| Vilar 2010 | 12 (0) | 9 | 12x self-reported improvement | Not reported | 3x dizziness / nausea | 0 | Not applicable | 12 | 9 /  12x self-reported improvement |

^a^N shows total number of patients with symptom at start study
